# Gradual changes within long-lived influenza virus-specific CD8^+^ T cells are associated with the loss of public TCR clonotypes in older adults

**DOI:** 10.1016/j.ebiom.2025.105697

**Published:** 2025-04-17

**Authors:** Carolien E. van de Sandt, Hayley A. McQuilten, Malet Aban, Thi H.O. Nguyen, Sophie A. Valkenburg, Emma J. Grant, Sneha Sant, Jamie Rossjohn, Stephanie Gras, Jane Crowe, Katherine Kedzierska

**Affiliations:** a Department of Microbiology and Immunology, University of Melbourne, at the Peter Doherty Institute for Infection and Immunity, Melbourne, VIC 3000, Australia; b WHO Collaborating Centre for Reference and Research on Influenza, Royal Melbourne Hospital, at the Peter Doherty Institute for Infection and Immunity, Melbourne, Australia; c HKU-Pasteur Research Pole, School of Public Health, The University of Hong Kong, Hong Kong SAR, China; d Infection and Immunity Program, Department of Biochemistry and Chemistry, La Trobe Institute for Molecular Science, La Trobe University, Bundoora, VIC 3086, Australia; e Immunity Program and Department of Biochemistry and Molecular Biology, Biomedicine Discovery Institute, Monash University, Clayton, VIC 3800, Australia; f Institute of Infection and Immunity, Cardiff University School of Medicine, Cardiff CF14 4XN, UK; g Deepdene Surgery, Deepdene, VIC 3103, Australia

**Keywords:** Longevity, Epitope-specific CD8^+^ T cells, TCR, Adults, Elderly, Influenza

## Abstract

**Background:**

Susceptibility to life-threatening influenza increases with age, partly due to declining immunity. Frequency, phenotype and T-cell receptor (TCR) composition of influenza-specific CD8^+^ T-cells directed at the prominent A2/M1_58_ influenza epitope change across the human lifespan.

**Methods:**

We investigated longevity and mechanisms underlying age-related changes in influenza-specific TCR repertoires by performing longitudinal analyses in young and older adults across 7–12 years within A2/M1_58_^+^CD8^+^ T-cells using peptide-HLA tetramers directly *ex vivo*. Paired TCRαβ-chains were used to track clonotypes over time within individuals.

**Findings:**

Expanded public and private TCR clonotypes were long-lived but gradually declined over time. Loss of public clonotypes was initially compensated by expansions of clonotypes expressing public-associated features. Once these public-associated TCR clonotypes were abated in older adults, the void was filled by expansions of less similar private TCR clonotypes. Expanded older private TCR clonotypes also declined over time and were gradually replaced by other private TCR clonotypes with low similarity to public TCR clonotypes detected in adults.

**Interpretation:**

Despite our relatively small cohort, we provided conclusive evidence that CD8^+^ T-cells to a single HLA-A2-restricted influenza-epitope are long-lived. However, dynamic changes occur at the clonotypic level, which eventually result in loss of public clonotypes, indicating that T-cell-based influenza vaccines are likely more effective in adults than older adults.

**Funding:**

This research was supported by the 10.13039/501100000925National Health and Medical Research Council (#1173871, #1159272), the 10.13039/501100000923Australian Research Council (#190102704), 10.13039/501100007601European Union’s Horizon 2020 (#792532), the 10.13039/501100001782University of Melbourne. Funders had no role in design, analysis or reporting of the study.

**Research in context**

**Evidence before this study**

CD8^+^ T cells, also known as killer T cells, play an important role in controlling viral infections by eliminating virus-infected cells. It is well established that the frequency and functionality of influenza-specific CD8^+^ T cells declines with age. However, in contrast to the existing dogma, we found that these changes did not result from CD8^+^ T cells becoming senescent or exhausted. Instead, influenza virus-specific CD8^+^ T cells expressing highly-functional public (shared) T cell receptors which were found in children and adults were replaced T cells expressing less-functional private (not-shared) T cell receptors in older adults. However, two fundamental questions remained: 1) whether influenza-specific CD8^+^ T cells are long-lived and 2) whether the change from public to private CD8^+^ T cells is a gradual change or an abrupt shift. These questions could only be answered using long-term longitudinal cohorts, with participants followed over time.

**Added value of this study**

We found that both young public and older private CD8^+^ T cells are long-lived, as they were consistently detected across multiple time points over a 12-year period, albeit they exhibited a gradual decline in over time. The initial decline of CD8^+^ T cells expressing public TCRs in adults was initially compensated by other closely related CD8^+^ T cells expressing very similar T cell receptor features. However, once these were ablated, the void was filled by the expansion of private CD8^+^ T clonotypes with distinct T cell receptor features.

**Implications of all the available evidence**

Our findings help explain why older adults are at higher risk for severe influenza disease outcomes. However, our data also demonstrate that novel CD8^+^ T-cell based influenza vaccines will likely be more effective in adults compared to older adults and warrants future monitoring when they become available.

## Introduction

Ageing is the largest cause of natural impaired immunity. By 2050, ∼22% of the world’s populations will be >65 years.^1^ Older adults are at high-risk of severe disease outcomes, particularly following respiratory viral infections during annual epidemics and sporadic pandemics.^2^^,^^3^ Yet, current vaccines and anti-viral treatment strategies generally provide suboptimal protection in older adults. Understanding the mechanisms underpinning waning of immune responses in older adults is of key importance if we are to rationally design improved vaccine and immunotherapy regimens to prevent life-threatening viral diseases in the ageing populations.

CD8^+^ T cells play an important role in clearing viral infections. As CD8^+^ T cells predominantly recognise conserved peptides derived from viral proteins in the context of HLA-I (human leucocyte antigen class I),^4^, ^5^, ^6^, ^7^, ^8^ they can be recalled even in the absence of antibodies when new viral variants or strains emerge,^4^^,^^9^^,^^10^ leading to rapid host recovery and milder disease.^11^^,^^12^ Our previous studies provided evidence for the importance of CD8^+^ T cells for protection against fatal influenza from novel IAVs^13^ and identified CD8^+^ T cells cross-reactivity across all influenza A, B and C viruses.^8^ T cells are also cross-reactive across SARS-CoV-2 variants of concern.^5^^,^^14^, ^15^, ^16^ One of the key hallmarks of virus-specific memory CD8^+^ T cells is their ability for a rapid recall, effective elimination of virus-infected cells, by which they contribute to the amelioration of disease severity.^13^^,^^17^, ^18^, ^19^ It is well documented that CD8^+^ T cells form long-lived immunological memory following infection or vaccination both in mice^20^, ^21^, ^22^, ^23^ and humans.^24^, ^25^, ^26^ Smallpox-specific T cells can be detected up to 75 years post vaccination, with an estimated half-life of 8–15 years.^27^ Persistence of virus-specific CD8^+^ T cell immunity in older adults has been, however, less studied at the clonal level.

Recently we defined CD8^+^ T cell immunity directed against one of the most prominent and conserved human influenza virus epitopes, HLA-A∗02:01-M1_58-66_ (A2/M1_58_) across the human lifespan.^28^ We demonstrated that the frequency, phenotype and T cell receptor (TCR) clonal composition of influenza-specific CD8^+^ T cells change as we age. While we identified a linear differentiation trajectory from newborns to children and then adults, there was a striking divergence in older adults. This discrepancy in older adults was associated with a clonal switch from influenza virus-specific public TCR clonotypes (shared between individuals) found in children and adults to suboptimal, private TCR clonotypes (specific to one individual) in older adults.^28^ The public signature of the younger TCRαβs, including those expressing public-associated CDR3 motifs, were highly functional and associated with higher proliferation capacity, polyfunctionality, higher avidity and better recognition of peptide variants. Conversely, suboptimal older private TCRαβ were less functional and associated with poorer proliferation, reduced polyfunctionality, avidity and less capable of recognizing peptide variants.^28^ Whether this age-specific shift in TCR clonotypes resulted from a gradual change in the TCR repertoire or represents a more abrupt clonal reset remains to be understood and requires longitudinal analyses of A2/M1_58_-specific CD8^+^ T cell populations within single individuals spanning multiple years. Previously, Naumova et al. performed a longitudinal study on *in vitro* A2/M1_58_ expanded TRBV19-expressing CD8^+^ T cell repertoires in middle-aged individuals across two timepoints, spanning 7–10 years, using the complementary-determining region 3 (CDR3) nucleotide sequence of single TCRβ-chain genes to study clonality.^25^ The authors found that influenza virus A2/M1_58_-specific CD8^+^ TCRβ clonotypes with a high frequency (observed multiple times across two time points) were relatively stable over time. However, public-associated TCRβ clonotypes expressing Arginine (R) and Serine (S) in their CDR3 region, a key motif associated with peg-notch docking mode,^29^ declined over time. Although this study corroborated findings observed in individuals across the human lifespan,^28^^,^^30^ some fundamental questions regarding the longevity and changes in the repertoire remain. Neither study was able to conclusively demonstrate at a molecular level whether a single clonotype, defined by a uniquely paired TCRαβ sequence, was indeed long-lived. It also remains unknown whether the clonal switch, observed in older adults, was related to a rapid clonal change or resulted from a gradual replacement of TCR clonotypes expressing closely-related public features to more distant private TCR clonotypes. Furthermore, we still do not understand whether this TCR clonal reset in older adults reflects a once in a lifetime event or results from a gradual clonal change within memory T cells which occur throughout the human lifespan. These questions can only be answered using longitudinal cohorts combined with paired TCRαβ analysis of antigen-specific CD8^+^ T cells.

Paired TCRα and -β chain sequences can be used as key barcodes to track A2/M1_58_-specific clonotypes over time within a single individual. In this study, we performed a longitudinal study of the paired TCRαβ repertoire of A2/M1_58_-specific CD8^+^ T cells directly *ex vivo* in a small cohort of three adults and four older adults over the course of 7–12 years. We demonstrate that the expanded public paired TCRαβ clonotypes in adults and expanded private paired TCRαβ clonotypes in older adults can be found continuously over course of 12 years. To the best of our knowledge, this is the first conclusive evidence that both public and private TCRαβ clonotypes are long-lived. However, both public and private TCRαβ clonotypes gradually decline over time, with a steeper decline observed in the older adults. Older TCR repertoires are more dynamic over time, with alternating large clonal expansions and TCR diversity across timepoints, explaining, at least in part, the loss of the influenza-specific public TCR clonotypes with age.

## Methods

### Study participants and ethics

Adults and older adults were recruited via the University of Melbourne (UoM; Melbourne, Australia) and Deepdene Medical Clinic (DMC; Deepdene, Australia). All participants provided informed written consent and were free of influenza symptoms at the time of sampling. Participants of the study did not receive any compensation. Peripheral blood mononuclear cells (PBMCs) were isolated using Ficoll–Paque (GE Health-Care, Uppsala, Sweden) gradient centrifugation, and cryopreserved in liquid N_2_ until required. HLA class I and II molecular genotyping was performed from genomic DNA by the Australian Red Cross Lifeblood (ARCL). Experiments conformed to the Declaration of Helsinki Principles and the Australian National Health and Medical Research Council Code of Practice. The study was approved by the Human Research Ethics Committee (HREC) of the University of Melbourne (Ethics ID #13344). Data from early time points for some donors was derived from previous studies: A2-2010 and A11-2010 were described in Valkenburg 2016.^31^ A2-2018, A11-2018, OA3-2019, OA18-2018, OA19-2018 and OA31-2019 were described in van de Sandt 2023.^28^ OA18-2013 and OA31-2019 were described in Nguyen 2017.^32^ A18-2012 (age 41) was described in Grant 2016^33^ and was the only donor timepoint that was measured after *in vitro* expansion of A2/M1_58_-specific CD8^+^ T cells following peptide stimulation of PBMCs. This study includes 8 new data points across 6 donors, namely A2-2022, A18-2022, OA3-2013 and 2023, OA18-2023, OA19 2013 and 2023 and OA31-2022 (Supplementary Table S1), these samples were processed as described below.

### Haemagglutination inhibition (HI) assay

The HI assay was performed by the World Health Organization Collaborating Centre for Reference and Research on Influenza in Melbourne Australia to measure antibody titres against influenza virus strains provided in Supplementary Fig. S1. Human plasma treated with receptor destroying enzyme (RDE) (Seiken RDE II, Denka Seiken Co., Ltd.) were serially diluted (two-fold) from 1:10 to 1:20480 in CaMg-free Phosphate Buffered Saline (PBS), pH 7.2 (Sigma–Aldrich) in a 96 well plate. Influenza viruses at 4HA units/25 μl were added to the diluted plasma and incubated for 1 h at room temperature. Turkey (for A/H1N1) or guinea pig (for A/H3N2) red blood cells (RBC) (1% v/v) were then added to the plasma/virus mixture and allowed to settle into distinctive patterns. The HI titre is the reciprocal of the highest dilution of the plasma inhibiting the agglutination of the RBC by the virus.

### *Ex vivo* A2/M1_58_ tetramer enrichment

PBMCs (1 × 10ˆ7) were thawed in complete RPMI (cRPMI) medium (RPMI1640 medium (Invitrogen) supplemented with 1 mM MEM sodium pyruvate (Gibco), 100 μM MEM non-essential amino acids (Gibco), 2 mM L-glutamine (Gibco), 5 mM HEPES buffer solution (Gibco), 55 μM 2-mercaptoethanol (Gibco), 100 U/ml penicillin (Gibco), 100 μg/ml streptomycin (Gibco) and 10% foetal bovine serum (Gibco)) and supplemented with 50 U/ml Benzonase (Novagen Merck) before tetramer-associated-magnetic enrichment (TAME) as previously described.^14^^,^^34^ Briefly, cells were resuspended in MACS buffer (PBS, 0.5% BSA and 2 mM EDTA) and magnetically enriched with PE-streptavidin conjugated A2/M1_58_ (GILGFVFTL) tetramer using anti-PE Microbeads (Milteny Biotec) and passed through an LS column (Milteny Biotec) to enrich A2/M1_58_ tetramer-positive cells. Cells were then surfaced stained in MACS buffer using anti-CD71-BV421 (1:50, BD Biosciences #562995), anti-CD3-BV510 (1:200, BioLegend #317332), anti-HLA-DR-BV605 (1:100, BioLegend 307640), anti-CD4-BV650 (1:100, BD Biosciences #563875), anti-CD27-BV711 (1:200, BD Horizon #563167), anti-CD38-BV785 (1:100, BD Biosciences #563964), anti-CD57-APC (1:400, BD Biosciences #560845), anti-CCR7-AF700 (1:50, BD Biosciences #561143), anti-CD14-APC-Cy7 (1:100, BD Biosciences #560180), anti-CD19 (1:100, BD Biosciences #560177), anti-CD45RA-FITC (1:200, BD Biosciences #555488), anti-CD8-PerCP-Cy5.5 (1:200, BD Biosciences #565310), anti-CD95-PECF594 (1:100, BD Horizon #562395), anti-PD1-1-PE-Cy7 (1:50, BD Biosciences #561272) and Live/Dead fixable aqua dead-cell stain (1:800, Invitrogen #L10119) and resuspended in MACS buffer for single cell-(index)-sorting using a BD FACSAria III (BD Biosciences), followed by the analysis using FlowJo software (v10.8.1) (BD Biosciences). All antibodies were validated for use on human cells by the manufacturers, and were titrated in our laboratory before use.

A2/M1_58_^+^CD8^+^ T cell frequencies were calculated relative to the total CD8^+^ T cell numbers in an unenriched fraction as described previously,^14^^,^^34^^,^^35^ using the following calculation

$$\frac{\#\;{Tetramer}^{+}\;{CD8}^{+}\;T\;cells\;in\;enriched\;fraction}{\left(Total\;counted\;lymphocytes\;in\;sample\times \%{CD8}^{+}\;T\;cells\;in\;unenriched\;fraction\right)\times 100}$$

The increase or decline relative to the first timepoint was calculated using the calculation

$$\frac{Frequency\;{Tetramer}^{+}{CD8}^{+}\;T\;cells\;timepoint\;1-Frequency\;{Tetramer}^{+}{CD8}^{+}\;T\;cells\;timepoint\;2\;or\;3}{Frequency\;{Tetramer}^{+}{CD8}^{+}\;T\;cells\;timepoint\;1}\times 100$$

Phenotypic populations were defined as T central memory (T_cm_; CD27^+^CD45RA^−^), T effector memory (T_em_; CD27^−^CD45RA^−^), terminally differentiated (T_emra_; CD27^−^CD45RA^+^), T stem cell memory (T_scm_; CD27^+^CD45RA^+^CD95^+^), T naïve (T_naïve_; CD27^+^CD45RA^+^CD95^-^) or T_naïve-like_ (CD27^+^CD45RA^+^) when no CD95 was included in the panel.

### Single-cell RT-PCR and paired TCRαβ sequencing

A2/M1_58_^+^CD8^+^ T cells were (index-) sorted into chilled 96-well twin.tec PCR plates (Eppendorf) and directly stored at −80 °C until required. Single-cell paired CDR3α and CDR3β regions were analysed by multiplex-nested RT-PCR and followed by sequencing of the CDRα and CDRβ products, as described previously,^31^^,^^34^^,^^36^ while using double amounts of reaction mix in the cDNA step for older samples. Sequences were analysed with FinchTV. V-J regions were identified by IMGT query (www.imgt.org/IMGT_vquest). TCR sequences were parsed using the TCRdist analytical pipeline.^37^^,^^38^ Clonotypes were defined as single-cell TCRαβ pairs that exhibit the same V, J and CDR3 regions.

### TCR repertoire analysis

Pairwise distances between TCRαβ clonotypes were determined using TCRdist,^37^ using the run_basic_analysis.py script available at https://github.com/phbradley/tcr-dist. This similarity measure was visualized using kernel principal component analysis (kPCA) calculated by TCRdist and plotted using ggplot2^39^ in the R computing environment (R version 4.2.1).^40^ Network graphs with edges connecting clonotypes ≤120 TCRdist units were generated in tcrdist3 (v0.2., Python v3.8.16)^38^ using the paired epitope-specific TCR workflow described in Mayer–Blackwell et al.,^41^ and exported to Cytoscape (v3.10.2)^42^ from R using igraph (v1.5.1),^43^ and RCy3 (v2.16.0) for formatting of network plots. CDR3 amino acid motifs and probability of generation (P_gen_) were determined using TCRdist^37^^,^^38^ with the run_basic_analysis.py workflow. Pairing of TCRαβ V-genes and public/private clonotypes over time were visualised using circlize (v0.4.16)^44^ in the R computing environment (R version 4.2.1).

### Statistical analysis

No statistical methods were used to pre-determine sample sizes but were instead determined by availability of HLA-A∗02:01 positive donors with repeated timepoints. Donors were stratified based on age. Experiments were not blinded, instead all analysis have been independently checked by multiple researchers. Experiments could not be replicated due to limited PBMC available. Normality tests were not performed, and non-parametric statistical analyses were performed in the study. Measurements were taken from distinct samples. Comparisons between timepoints were analysed by GraphPad Prism (v9.3.0, Graphpad) using a two-sided Mann–Whitney U-test for comparisons between two timepoints and a two-sided Kruskal–Wallis test combined with Dunn’s correction for multiple tests when comparing among three timepoints, unless otherwise indicated. A linear mixed effects model was used to assess the relationship between the frequency of isolated clonotypes and TCRα and TCRβ generation probability, while accounting for repeated measurements from the same individual. *p*-values were obtained based on the standard error of the estimated parameters in the model. The model was fitted using the nlme library in R (v4.3.0). Differences were considered significant at *p*-value of <0.05. Simpson’s diversity index (SDI) was calculated as previously described.^45^ Data collection and analysis were not performed blind to the conditions of the experiments.

### Code availability

No custom code or mathematical algorithms were used in the preparation of the manuscript.

### Role of the funding source

Funding sources did not have any role in the study design, data collection, data analysis, interpretation of the data, in writing the report, or in the decision to submit the manuscript for publication.

## Results

### Longitudinal HLA-A∗02:01^+^ cohort

**Fig. 1:**
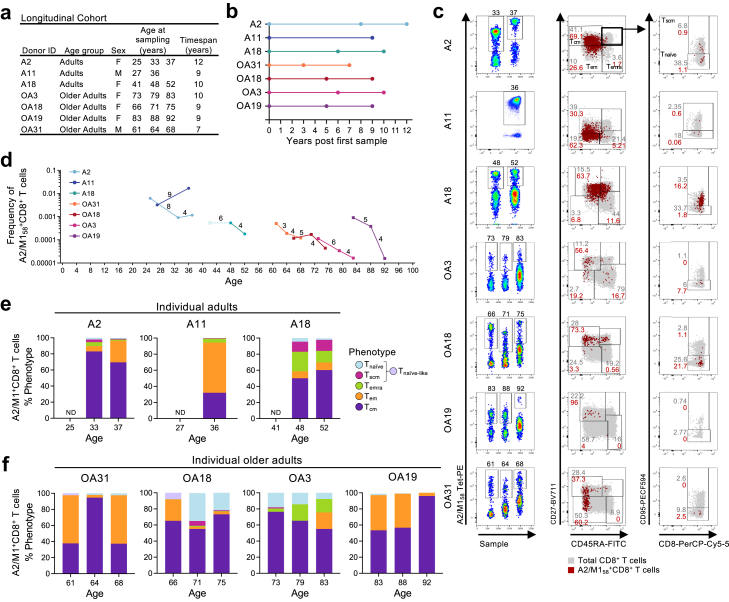
**Longitudinal changes in A2/M1_58_^+^CD8^+^ T cell frequencies and phenotypes. a)** Longitudinal HLA-A∗02:01 positive cohort and age at time of sampling. **b)** Years post first timepoint. **c)** Representative FACS panels and gating strategy for enriched A2/M1_58_^+^CD8^+^ T cells and their phenotypic characteristics: T_cm_ (CD27^+^CD45RA^−^) cells, T_em_ (CD27^−^CD45RA^−^), T_emra_ (CD27^−^CD45RA^+^), T_scm_ (CD27^+^CD45RA^+^CD95^+^) and T_naïve_ (CD27^+^CD45RA^+^CD95^−^) or as T_naïve-like_ (CD27^+^CD45RA^+^) cells when no CD95 was included in phenotypic panel. Total CD8^+^ T cells in the unenriched sample are in grey, A2/M1_58_^+^CD8^+^ T cells from the enriched sample in red. Numbers above the left FACS panels represent the age of the donor at time of sampling. **d)** Changes in A2/M1_58_^+^CD8^+^ T cell frequencies over time within each individual. A2/M1_58_^+^CD8^+^ T cell frequency for the first timepoint of A18 could not be established and was plotted as an open symbol and dotted line. The number of years between two timepoints had been indicated above each connecting line. Phenotypes of A2/M1_58_^+^CD8^+^ T cells in **e)** individual adult and **f)** older adult donors. No statistical analysis was performed for **(e)** and **(f)** as each bar represents a phenotype frequency for a single donor timepoint.

We defined TCR repertoires within influenza-specific A2/M1_58_^+^CD8^+^ T cells in a longitudinal cohort of healthy HLA-A∗02:01-expressing individuals across two immunologically-distinct age groups: adults (n = 3, 25–52 years) and older adults (n = 4, 61–92 years) across 2–3 time-points over the course of 7–12 years (Fig. 1a and b, Supplementary Table S1). We performed *ex vivo* phenotypic and paired TCRαβ repertoire analysis for circulating A2/M1_58_-specific CD8^+^ T cells. Serology performed between the first and second timepoint of each donor revealed potential infections for donor A2 (H3N2, around 2012), A18 (H3N2, around 2012), OA18 (H3N2, around 2014), OA3 (pH1N1, year unknown). No interim infections were reported for A11, OA31 and OA19. Interpretation is based on the virus to which the highest titre was detected (Supplementary Fig. S1a). However, it is important to note that influenza antibody HI titres do not differentiate between influenza virus infection and vaccination.

### Frequency of A2/M1_58_-specific T cells declines with increasing age

Tetramer-associated-magnetic enrichment (TAME)^14^^,^^28^^,^^34^^,^^46^ was used to probe the magnitude and phenotype of A2/M1_58_^+^CD8^+^ T cells directly *ex vivo* (Fig. 1c–f, Supplementary Fig. S1b). Overall, frequencies of A2/M1_58_^+^CD8^+^ T cells declined across the years for 6 out of 7 donors (median decline 78.62%, range 62.65%–98.27%), with the exception of A11 who had a 5.25-fold increase. A decline in A2/M1_58_^+^CD8^+^ T cells was more evident in older adults (median 80.76%) compared to adults (median 66.7%), albeit not significant (Fig. 1d). This may align with reports of declining CD8^+^ T cell populations with increasing age.^28^^,^^32^^,^^47^^,^^48^

### Dynamic phenotypic changes across timepoints

Phenotypic analysis was performed directly *ex vivo* using CD27, CD45RA and CD95 to identify A2/M1_58_^+^CD8^+^ T central memory (T_cm_; CD27^+^CD45RA^−^), T effector memory (T_em_; CD27^−^CD45RA^−^), terminally differentiated (T_emra_; CD27^−^CD45RA^+^), T stem cell memory (T_scm_; CD27^+^CD45RA^+^CD95^+^), T naïve (T_naïve_; CD27^+^CD45RA^+^CD95^-^) or T_naïve-like_ (CD27^+^CD45RA^+^) when no CD95 was included in the panel. No phenotypic anlysis was performed for the first timepoint of the adult donors A2, A11 and A18 (Fig. 1c,e, and f, Supplementary Fig. S1b). Although adult A2/M1_58_^+^CD8^+^ T cell populations were heterogenous between individual adults, they appeared stable across the measured timepoints in A2 and A18. Adult A2/M1_58_^+^CD8^+^ T cell populations were defined by a prominent T_cm_ phenotype (A2), prominent T_em_ phenotype (A11) or mixed memory (T_cm_ > T_emra_ > T_scm_ > T_em_) phenotype (A18) (Fig. 1e). The phenotype of A2/M1_58_^+^CD8^+^ T cell in older adults were also heterogeneous between donors, but more prominent phenotypic changes were observed across timepoints within individual donors (Fig. 1f). The A2/M1_58_^+^CD8^+^ T cells in OA31 and OA19 displayed a prominent mixed T_em_ > T_cm_ phenotype in 2 of 3 timepoints, with the remaining timepoint displaying a spike in T_cm_ cells. The A2/M1_58_^+^CD8^+^ T cells of OA18 and OA3 were predominantly T_cm_ across all three timepoints, although the T_cm_ frequency tended to decrease over time in OA3, while T_em_ and T_emra_ populations modestly increased (Fig. 1f).

Despite the small cohort size we demonstrate that the A2/M1_58_-specific CD8^+^ T cell repertoire is characterized by a prominent T_cm_/T_em_ phenotype across all timepoints. It is likely that the time-specific changes in older donors may have resulted from recent infections as previous observed.^24^

### Distinct stable and dynamic clonotypes in adult and older TCR repertoires over time

**Fig. 2:**
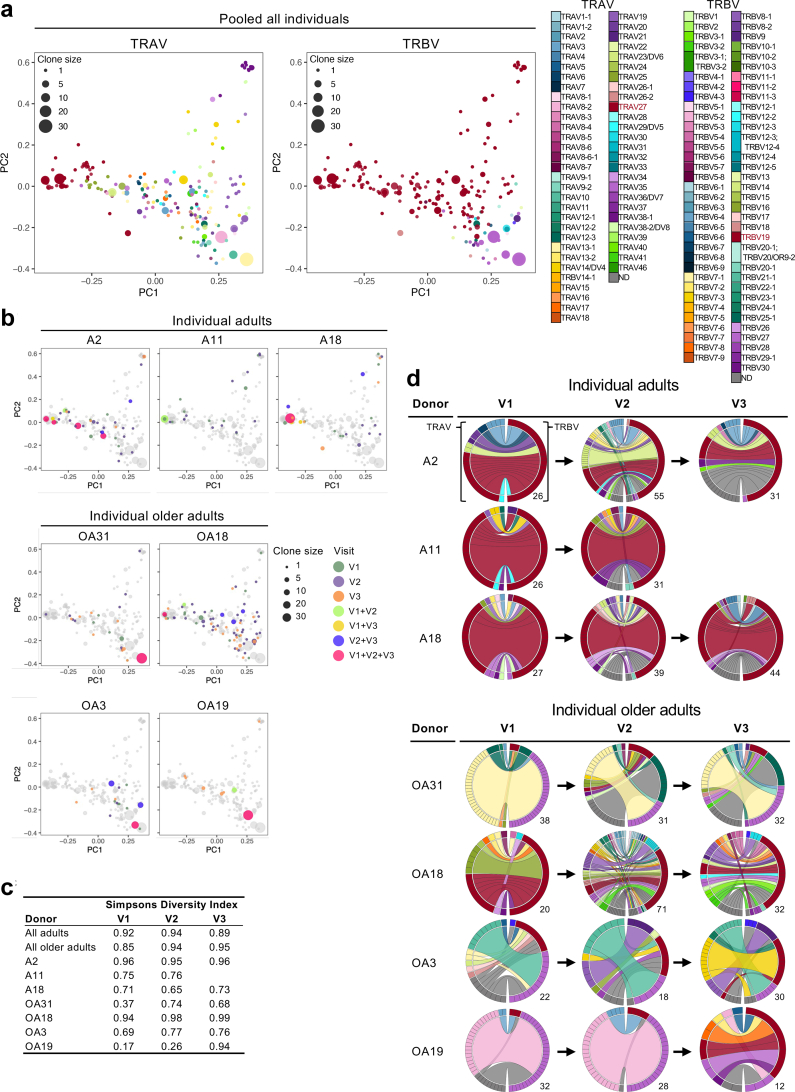
**Dynamic changes in the A2/M1_58_^+^CD8^+^ TCRαβ repertoire.** Enriched A2/M1_58_^+^CD8^+^ T cells were single-cell sorted for TCRαβ analysis. TCRdist generated 2D kernel principal components analysis (kPCA) projection of the pooled A2/M1_58_^+^CD8^+^ TCR landscape for all donors combined (n = 7) coloured by **a)** Vα (left), and Vβ (right) gene usage. **b)** 2D kernel PCA projection of the A2/M1_58_^+^CD8^+^ TCR landscape coloured by timepoint (visit) of individual donors, the full repertoire of all donors combined is indicated in grey at the background. **a and b)** Clone size reflective of the count across all the time points is indicated by symbol size. **c)** Longitudinal changes in Simpson’s Diversity Index scores for pooled adults (V1 and V2 n = 3, V3 n = 2), pooled older adults (V1–V3 n = 4) or individual donors. Samples for pooled groups were combined prior to calculating the SDI. No statistical differences were found due to the small sample size. **d)** TRAV and TRBV clonotype pairing for individual donor across timepoints illustrated by circos plots. Left arch segment coloured by TRAV usage, right outer arch coloured by TRBV usage. Connecting lines indicated TRAV-TRBV gene pairing coloured based on their TRAV usage and segmented based on their CRD3α and CDR3β sequence, the thickness of each connecting segment is proportional to the number of TCR clones with the respective pair. The number of sequences for each circos plot are shown at the right bottom.

**Fig. 3:**
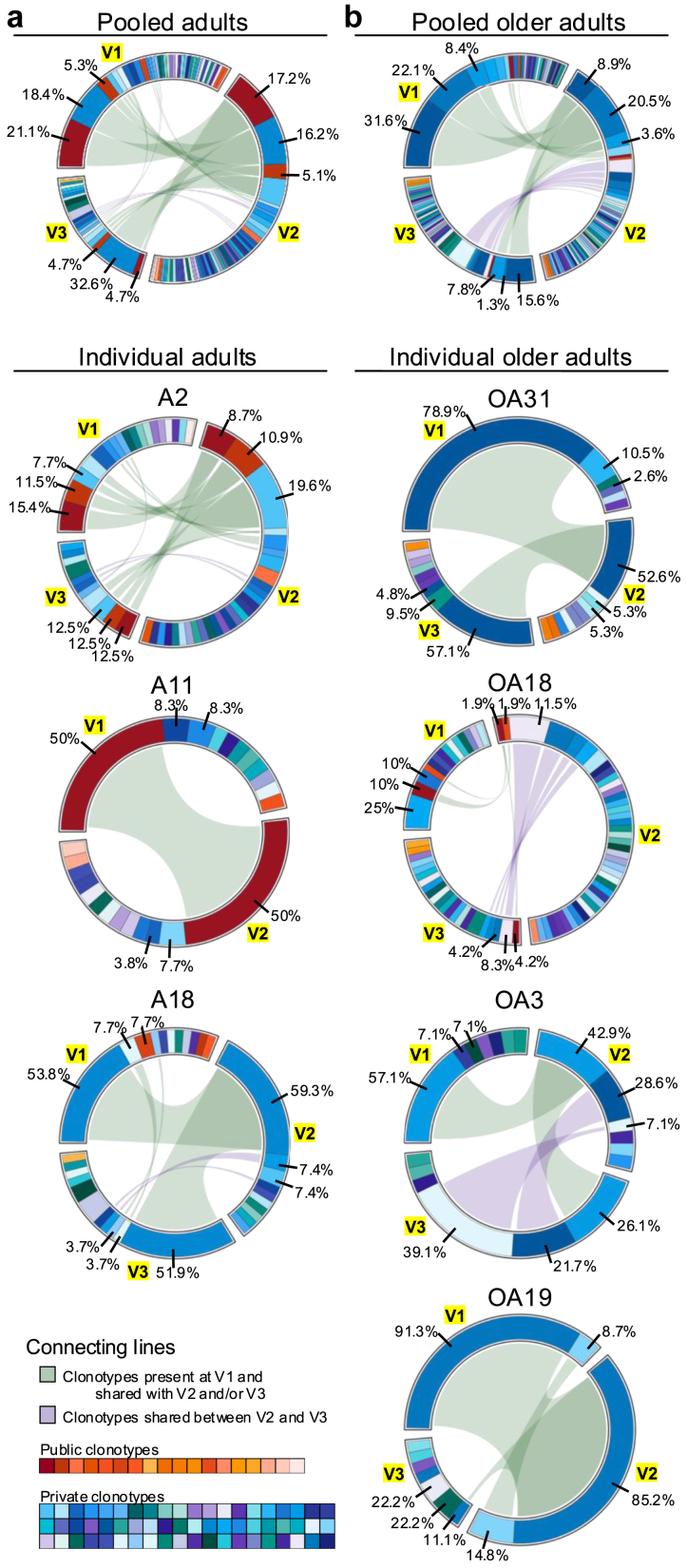
**Longitudinal changes in public and private A2/M1_58_^+^CD8^+^ TCRαβ clonotypes.** Frequency of previously defined public (shared, warm colours) and private (not shared, cool colours) clonotypes^28^ across the three timepoints (V1, V2, V3) for **a)** pooled (n = 3) and individual adult donors and **b)** pooled (n = 4) and individual older adult donors. Connecting lines represent clonotypes present at V1 and shared with V2 and/or V3 (green) or clonotypes present at V2 shared with V3 (purple). Percentages for top three clonotypes in V1 indicated, if not shared between timepoints, the percentage for the next most prevalent sequences in subsequent timepoints is indicated.

**Fig. 4:**
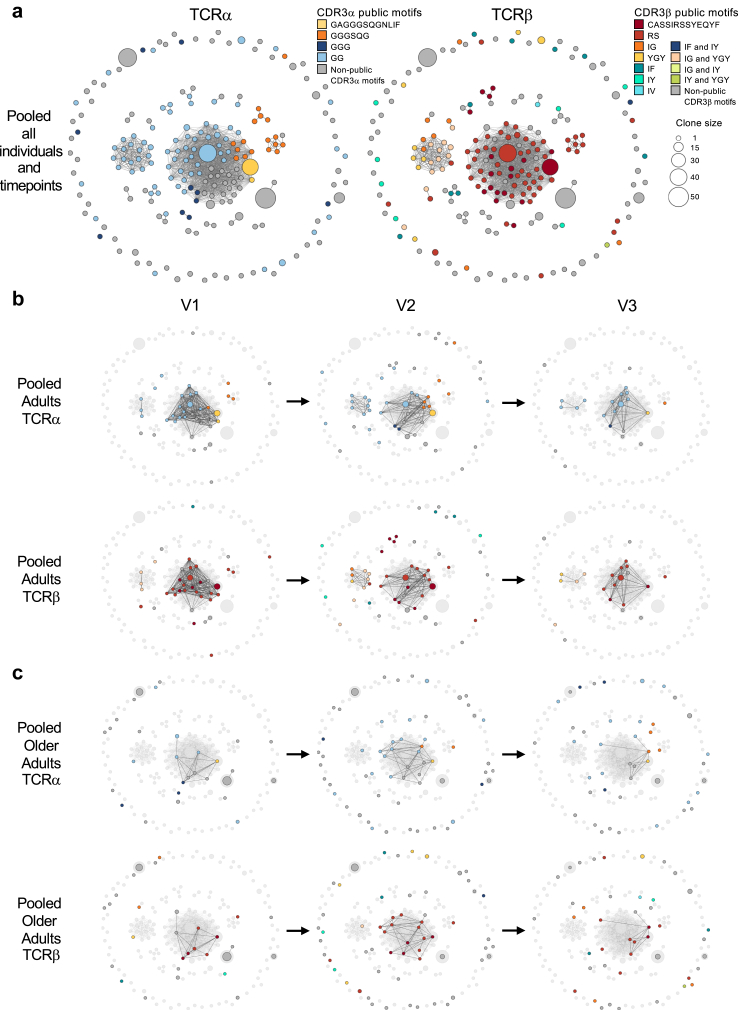
**High similarity A2/M1_58_^+^CD8^+^ TCRαβ clonotypes decrease with age. a)** A2/M1_58_-specific TCRαβ clonotypes feature clusters of highly similar sequences of all individuals combined (n = 7) and timepoints (n = 3) pooled. Each node of the similarity network is a uniquely paired TCRαβ sequence, edges connect TCRαβ with TCRdist equal to or less than 120. Coloured by TCRα-motifs (left) and TCRβ-motifs (right). Single clonotypes without connections are depicted in the outer circle. A2/M1_58_-specific TCRαβ clonotypes of **b)** pooled adults (n = 3) and **c)** pooled older adults (n = 4), split by timepoint. Coloured by TCRα-motifs (top) and TCRβ-motifs (bottom), larger network **(a)** depicted in light grey. Clusters for individual donors and timepoints can be found in Supplementary Fig. S4.

**Fig. 5:**
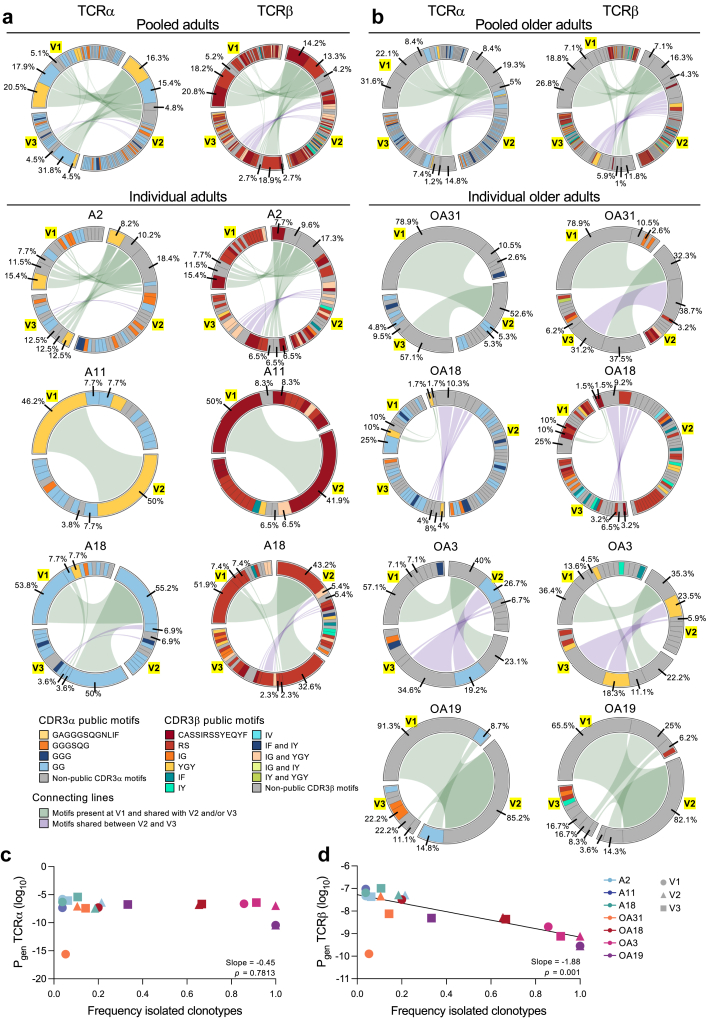
**Declining P_gen_ underpins loss of public-associated CDR3αβ motifs and similarity within older A2/M1_58_^+^CD8^+^ TCR repertoires.** Persistence of TCR clonotypes expressing selected prominent CDR3α- (left) and CDR3β- (right) motifs^28^ in **a)** pooled adults (n = 3) and individual adult donors and **b)** pooled older adults (n = 4) and individual older adult donors across three timepoints (V1, V2, V3). Colours identify the most prominent CDR3α and CDR3β-motifs. Shared clonotypes are connected by coloured lines. Connecting lines represent clonotypes present at V1 and shared with V2 and/or V3 (green) or clonotypes present at V2 shared with V3 (purple). Percentages for top three clonotypes in V1 indicated, if not shared between timepoints, the percentage for the next most prevalent clonotype in subsequent timepoints is indicated. Correlation between the frequency of low similarity single clonotypes in Fig. 4 with the probabilities of generation (P_gen_; log10 transformed) for all single **c)** TCRα and **d)** TCRβ chains of each donor and timepoint estimated with TCRdist.^37^ Correlation was established by using a linear mixed effects model, slope and *p*-values are indicated in the right bottom corner of the graph.

The diversity and clonal composition of the TCRαβ repertoire affects CD8^+^ T cell functionality and protection.^28^^,^^34^^,^^49^^,^^50^ To define whether influenza virus-specific CD8^+^ T cells are long-lived and to understand whether the shift from a public TCR-dominated repertoire in adults to a private TCR repertoire in older adults is a gradual, abrupt or recurrent phenomenon, we dissected the paired TCRαβ clonal diversity and composition of A2/M1_58_-specific CD8^+^ T cells in our donors directly *ex vivo* across 2–3 timepoints (Fig. 2, Fig. 3, Fig. 4, Fig. 5, Supplementary Figs. S2–S6 and Table S2). We analysed 502 paired A2/M1_58_^+^CD8^+^ TCRαβ clonotypes, 19 single TCRα and 124 single TCRβ chains with an unidentified matching TCRβ or TCRα chain (Supplementary Fig. S2a and Table S2).

2D kernel principal components analysis (kPCA) projection of the TCR Vα, Vβ, Jα and Jβ gene segment landscapes are a useful tool to illustrate relationships between individual paired TCRαβ within the antigen-specific TCR repertoire. Clonotypes who share TCR characteristics, including gene usage, will cluster together in the landscape, whereas TCRs with more unique characteristics will appear as outliers in the projection. The kPCA landscapes displayed high diversity within the TCR repertoire of the whole cohort^37^ (Fig. 2, Supplementary Fig. S2b–d). Public-associated TRBV19- and TRAV27-expressing clonotypes were prominently featured within the TCR landscapes (Fig. 2a).^28^ Paired TRAV-27-TRBV19 expressing clonotypes were predominantly found on the left side of the kPCA plot (Supplementary Fig. S2b). The TCR repertoires of 3 adults (A2, A11 and A18) were dominated by TRBV19-expressing clonotypes which could be identified across all 3 timepoints, with large clonal expansion on the left side of the graph indicating TRAV27-TRB19 expressing clonotypes that could be detected across all timepoints (Fig. 2b, Supplementary Fig. S2c and Table S2). TRAV gene usage, other than TRAV27, was also observed in adult donors, particularly in donor A2. The only older adult expressing several TRAV27-TRBV19 expressing clonotypes across the different time points was OA18 (Fig. 2b, Supplementary Table S2), yet we observed that more distant clonotypes became more prominent over time (Fig. 2b, Supplementary Fig. S2d). Older adults OA31, OA3 and OA19 clustered more to the right and their TCR repertoire was dominated by TRBV27-expressing clonotypes which were identified across all 3 timepoints (Fig. 2b, Supplementary Fig. S2d and Table S2). Diversity within adult donors and across timepoints were mainly affected by changes in TRAV usage, whereas both the TRAV and TRBV gene segments contribute to diversity in the TCR repertoire of older adults (Fig. 2b, Supplementary Fig. S2c and d).

The Simpsons Diversity Index (SDI) is a measure of diversity, which considers the number of TCRs and the relative abundance of each TCR within the repertoire, with a value closer to one indicating greater repertoire diversity. Although the overall SDI in adult and older adult TCR repertoires was relatively similar, the adult SDI slightly decreased from 0.92 at the first timepoint (V1) to 0.89 by the third timepoint (V3). Conversely, the SDI in older adults appeared to increase from 0.85 (V1) to 0.95 (V3) (Fig. 2c). SDI in two out of three adults (A11 and A18) ranged between 0.65 and 0.76 across timepoints, with greater variation between timepoints observed in A18. The SDI in A2 was stably high across all three timepoints (range 0.95–0.96). The lowest SDI scores were observed at the first timepoint of OA19 (0.17) and OA31 (0.37), both increasing over time to 0.94 (OA19 V3) or 0.74 (OA31, V2) after which SDI dropped to 0.68 by V3 (Fig. 2c). The SDI for OA3 was within a similar range to those observed in adult A11 and A18, with a slight increase in SDI from 0.69 (V1) to 0.76 (V3). In contrast, OA18 started with a high SDI of 0.94 (V1) which modestly increased to 0.99 (V3) (Fig. 2c).

Taken together, our data suggest that the overall TCR diversity of the adult TCR repertoire is relative stable across time points. In contrast, heterogenic changes in the TCR repertoire diversity were observed in older adults across timepoints, including highly restricted repertoires which diversify again over time. Diversity within the adult TCR repertoire mainly results from broader TCRα gene usage, whereas both the TCRα and TCRβ chain contribute to diversifying the older TCR repertoire, which is in line with our previous findings.^28^

### Variation in gene segment usage in older TCR repertoires over time

To understand whether changes in gene segment usage underpin the changes in TCR repertoire diversity, we performed circos analyses of individual TRAV and TRBV sequences. TRBV19-TRAV27-expressing clonotypes dominated the adult TCR repertoires over time (Fig. 2d, Supplementary Fig. S2c and Table S2). Minor variations in TRAV usage were observed across all three timepoints for all three adult donors. TRBV19-TRAV27-expressing TCR clonotypes were found in all three timepoints of older adult OA18, but sharing became less prevalent over time (Fig. 2d, Supplementary Table S2). TRBV19-TRAV27-expressing TCR clonotypes were also detected in the last timepoint (V3) in OA19 and in a single clonotype at V2 in OA31, but were not observed in OA3 (Fig. 2d). In contrast, the older TCR repertoire was dominated by large clonal expansions of clonotypes expressing TRBV27 paired with TRAV13-1 (OA31), TRAV10 (OA3) or TRAV8-2 (OA19) (Fig. 2d, Supplementary Fig. S2d and Table S2). Overall, more prominent differences in TRAV and TRBV genes were observed across timepoints in all four older adults.

These results are in agreement with our recent findings which demonstrated that public-associated clonotypes (clonotypes expressing public features including TRAV27 and/or TRBV19) disappear with increasing age.^28^

### Young public and older private clonotypes are long-lived but gradually decline over time

To determine whether changes in the variable gene segments affect the abundance of public and private clonotypes, we defined the distribution of public and private clonotypes across timepoints directly *ex vivo* (Fig. 3, Supplementary Table S2). Public clonotypes are defined as clonotypes shared among individuals in our larger human lifespan cohort (more frequently observed in children and adults and were previously shown to have higher functional properties),^28^ whereas private clonotypes are not-shared among individuals in our larger human lifespan cohort (more abundant in older adults and were shown to have lower functionality).^28^ The frequency of public clonotypes decreased in the pooled adults over time, particularly in the previously described full public TCRαβ clonotype (TRAV27, TRAJ42, CDR3α-GAGGGSQGNLIF, TRBV19, TRBJ2-7, CDR3β-CASSIRSSYEQYF) associated with optimal immunity in adults,^28^^,^^29^^,^^31^^,^^51^^,^^52^ which trended to decrease from 21.1% at V1 to 17.2% at V2 and 4.7% at V3 (dark red in Fig. 3a; clone A in Supplementary Fig. S3 and Table S2). However, there was heterogenicity within the full public TCR-expressing clonotypes across individual adult donors. Donor A11 maintained their public TCR over the course of 9 years, whereas the full public TCR in donor A2 slightly decreased from 15.4% at V1 to 8.7% in V2 and slightly increased to 12.5% 12 years later at V3. Public clonotypes in donor A18 were scarce and not shared between timepoints. Instead, the TCR repertoire of A18 was dominated by a large private TRAV27, TRBV19 expressing clonotype across all 3 timepoints (Figs. 2d and 3a, clone CF in Supplementary Fig. S3 and Table S2). Of note, this non-public clonotype in donor A18 did express the public-associated CDR3 motifs (CDR3α-GG and CDR3β-RS) (clone CF in Supplementary Table S2). Public clonotypes became scarcer in the older adult TCR repertoires, with limited sharing across timepoints. The only older adult expressing a public clonotype across all 3 timepoints was OA18, with frequencies decreasing from 10% at V1 to 4.2% at V3 (dark red in Fig. 3b; clone A in Supplementary Fig. S3 and Table S2). However, expanded private clonotypes dominating the older TCR repertoire also appeared to decrease over time, with two most prominent private clonotypes decreasing from 31.6% to 22.1% at V1 to 15.6% and 1.3% at V3 respectively (Fig. 3b, clone DZ and JP in Supplementary Fig. S3 and Table S2). This trend mirrored observations in all 4 older adults, where the most prominent private clonotype at V1 became less abundant by V3 in OA31, OA3 and OA19 or was not shared at all between timepoints (OA18). In two out of four older adults (OA18 and OA3) another private clonotype became more prominent (Fig. 3b, OA18 clone FL, OA3 clone JC in Supplementary Fig. S3 and Table S2).

Public and private clonotypes can be detected over the course of 7–12 years, demonstrating their longevity. Overall, these data demonstrated that adult clonotypes appear more consistent across timepoints compared to clonotypes in older adults. However, dynamic changes in variable gene segments were observed in both adults and older individuals over time, which resulted in a gradual decline of expanded public TCR clonotypes in adults and expanded private TCR clonotypes in older adults.

### Lack of public CDR3 features in older TCR repertoires associated with a decrease in TCR similarity

The hypervariable CDR3 regions of TCRα and TCRβ chains mediate fine pHLA-I specificity. We recently discovered that TCRs expressing public-associated CDR3 motifs (motifs that are found across multiple donors, include CDR3α-GAGGGSQGNLIF, GGGSQG, GGG, GG, and/or CDR3β-CASSIRSSYEQYF, RS, IG, YGY, IF, IY, IV) are linked to superior immunity in children and adults and disappear in the older population.^28^ We therefore investigated whether the level of TCR sequence similarity and dynamic changes in the TCR repertoire over time were associated with changes in the CDR3 motifs. Hereto, we constructed a similarity network of paired, unique TCRαβ sequences (Supplementary Table S2), using a threshold of 120 TCRdist units on the TCRdist^37^^,^^38^ similarity measure to identify highly similar clonotypes (Fig. 4a–c, Supplementary Fig. S4). A similarity network based on all paired TCRαβ sequences in our dataset, revealed that clusters of similar sequences with two or more members displayed a striking positional enrichment of public-associated CDR3β motif and closely related public-associated CDR3α motifs (inner clusters Fig. 4a). Public-associated CDR3α and CDR3β motifs were in lower abundance in TCRs with low similarity to other TCRs in the network (outer ring Fig. 4a).

As the next step, we investigated whether the gradual change from public TCRs in adults to private TCRs in older adults^28^ was linked to changes in TCR similarity over time. Highly prevalent adult clonotypes expressing public-associated CDR3α and CDR3β motifs were prominently featured and shared across timepoints in adults. Although public-associated CDR3α and CDR3β motifs were also observed among TCRs with low similarity, they were not shared between timepoints of adults (Fig. 4b, Supplementary Fig. S4). Expanded clonotypes expressing public CDR3α and/or CDR3β motifs in V1 became less abundant in the third timepoint (A2 and A18) and were replaced by closely-related clonotypes which were shared between V2 and V3 (Fig. 5a, Supplementary Figs. S4–S6 and Table S2). Conversely, highly similar clonotypes expressing public-associated CDR3α and CDR3β motifs were less prominent in older adults but had the potential to be shared across timepoints, as observed in OA18, OA3 and OA19 (Figs. 4c and 5b, Supplementary Figs. S4–S6 and Table S2). Public-associated CDR3β motifs were more frequently observed among older TCRs with low similarity, and were shared between timepoints. In addition, expanded clonotypes with low similarity and private CDR3α and CDR3β motifs were also shared between timepoints (Figs. 4c and 5b, Supplementary Figs. S4–S6 and Table S2). OA18 was the only older adult to share the full public clonotype between three points, albeit at low and decreasing frequency (Fig. 5b, Supplementary Fig. S6, clone A in Supplementary Table S2). Another, CDR3β “RS”-expressing clonotype with a non-public CDR3α was shared between V2 and V3 and appeared to decrease in frequency (Fig. 5b, Supplementary Fig. S6, clone FM in Supplementary Table S2). OA3 shared an expanded clonotype with low similarity to other TCRs expressing the public-associated CDR3α “GG” motif and the CDR3β “YGY” motif between V2 and V3, which trended to decrease in frequency over time (Fig. 4, Fig. 5b, Supplementary Figs. S4 and S6, clone JB in Supplementary Table S2). OA19 shared a CDR3α “GG” expressing clonotype of low similarity across V1 and V2 with increasing frequency, which subsequently disappeared in V3 (Fig. 4, Fig. 5b, Supplementary Figs. S4 and S6, clone JR in Supplementary Table S2). Clonotypes expressing public-associated CDR3 motifs were not shared among timepoints in OA31 (Fig. 4, Fig. 5b, Supplementary Figs. S4 and S6, Supplementary Table S2).

Our findings support the hypothesis that a decline of public clonotypes in adults is compensated by expansion of other highly similar TCR clonotypes expressing public CDR3α and CDR3β motifs which are associated with strong avidity for the A2/M1_58-66_ epitope.^28^ The lack of public-associated CDR3α and CDR3β motifs in the older TCR repertoire, results in expansion of low similar clonotypes expressing private CDR3α and CDR3β motifs, associated with lower avidity for the A2/M1_56-66_ CD8^+^ T cell epitope.^28^

### Low probability of generation underpins low similarity within older A2/M1_58_-specific TCR repertoires

The gradual shift from public to private clonotypes may result from changes in TCR recombination during thymic development. New antigen-specific clonotypes emerge from the thymus. However, gradual thymic involution starts during childhood and ceases after the fourth decade of life, hampering replenishment of naïve T cells and thereby reducing circulating pools of naïve T cells.^53^ These changes may not only affect thymic output but also directly affect the TCR recombination process, and thus the probability of generation (P_gen_) of the TCRs within the circulating repertoire.^28^ Clonotypes with larger P_gen_ values are easier-to-generate compared to clonotypes with smaller P_gen_ values, which are harder-to-generate and therefore rarer. We previously demonstrated that the public A2/M1_58_-specific clonotypes shared among younger individuals were easier-to-generate, as demonstrated by higher P_gen_ of their TCRβ chain.^28^ We estimated the P_gen_ of the A2/M1_58_^+^CD8^+^ TCRα and -β chains using TCRdist^37^ and confirmed that the P_gen_ of the TCRβ but not the TCRα chain negatively correlated with age (Supplementary Fig. S7a). This trend was mirrored in the 9–10 year timeframe of older adult donors OA18 and OA3, but no such correlation was identified among the 7–12 year timeframe of individual adult donors, albeit the Pgen of OA19 was lower as those observed in the adult donors across all 3 timepoints (Supplementary Fig. S7b and c).

To understand whether reduced P_gen_ of A2/M1_58_-specific TCRαβ was associated with the observed decrease in similarity of TCRs, we correlated the P_gen_ against the frequency of low similarity clonotypes within the TCR repertoire of the donors in our longitudinal cohort across different timepoints. The P_gen_ of TCRβ chains negatively correlated with the frequency of low similarity TCR clonotypes which dominated the repertoire in older adults (Fig. 5d). No correlation was observed for the TCRα chains (Fig. 5c).

These data demonstrate that the observed decrease in the TCRβ P_gen_ is associated with a loss of public-associated CDR3 motifs and a decrease in TCR similarity within the A2/M1_58_-specific TCRαβ repertoire of older adults over time.

## Discussion

Age is associated with a decline in immune functions, also known as immunosenescence, impacting both humoral and cellular immunity following infections and vaccination.^28^^,^^54^ Some of the hallmarks of immunosenescence include 1) reduced production of high-affinity antibodies by B cells,^55^ which play an important role in neutralizing viruses and preventing infections, 2) thymic involution results in decreasing populations of naïve T cells, decreasing the potential to recognize novel pathogens^56^ and 3) the continuous exposure to pathogens throughout human life results in the accumulation of memory T cells, including senescent and exhausted T cells with reduced capacity to expand and mediate functions, making them less effective against pathogenic invaders. However, we recently demonstrated that influenza virus-specific cells in older adults lack the classical senescent and exhaustion characteristics.^6^^,^^28^ Instead, influenza virus-specific CD8^+^ T cells shifted from highly functional TCR in children and adults to suboptimal TCRs in older adults.^28^ These findings explain, at least in part, why older adults have diminished capacity to respond to previously encountered and novel pathogens, control chronic infections and tumour suppression and why vaccinations become less effective with age.^2^^,^^54^^,^^57^, ^58^, ^59^, ^60^

Our study assessed a fundamental question about the longevity of influenza virus-specific CD8^+^ T cells in adults and older adults, and provides a potential explanation for the influenza-specific TCR switch observed in older adults. Using our in-depth pipeline to analyse paired TCRαβ clonotypes, we were, to the best of our knowledge, the first to conclusively demonstrate that both public TCR clonotypes in adults and private TCR clonotypes in older individuals are long-lived, as they were continuously detected within a single individual over the course of 7–12 years, although frequencies declined over time. Using our unique longitudinal cohort of adult and older adult donors, we corroborated previous findings by us^28^ and others,^25^^,^^30^ that clonotypes expressing public CDR3α and CDR3β motifs are preferentially selected upon declining frequencies of the full public TCR clonotype. This was most clearly demonstrated in A18, where lack of the full public clonotype resulted in clonal expansion of a highly similar private TCR clonotype expressing public-associated CDR3α and CDR3β features which was maintained over time. Furthermore, we not only confirmed our earlier finding that P_gen_ declined with increasing age,^28^ but additionally demonstrated that this decline underpins the diversity within the older TCR repertoires. Together our findings indicate that the clonotype switch from adults to older adults, observed in our previous study,^28^ resulted from a gradual decline of public clonotypes in adults, which was initially compensated by expansion of clonotypes expressing public-associated features. Once these public-associated TCR clonotypes were abated in older adults, the void was filled by expansions of private TCR clonotypes, which shared less similarity with young clonotypes expressing public features. Expanded private clonotypes in older adults also declined over time and were gradually replaced by other private clonotypes with low similarity to the public clonotypes observed in adults (Supplementary Fig. S8).

The cause of the decline of both public and private clonotypes over time remains to be established, although it is likely that a combination of factors plays a role. Repeated influenza infections experienced over the course of the human lifespan may result in the expansion and subsequent contraction of the clonotypes with the highest functional properties, including binding avidity and functionality, which is in line with our previous findings,^24^^,^^28^ whereas the parallel thymic involution^53^ may skew the repertoire into the direction of private TCR clonotypes.^28^ However, the sharp decline in A2/M1_58_-specific CD8^+^ T cells observed between V2 and V3 in 4 out of 7 donors, may have been augmented by the low prevalence of seasonal influenza during the COVID-19 pandemic years.^61^ McMichael et al. observed a similar steep decline following several low prevalent influenza seasons between 1978 and 1982.^48^

We previously found that peaks in influenza-specific CD8^+^ T cell frequency and effector memory phenotypes, over the course of 13 years, were associated with recent infections prior to collection of the respective blood sample.^24^ Although we observe similar changes in our current cohort, these could not conclusively be linked to recent infections, as donors in our cohort likely received annual vaccination, resulting in inconclusive antibody detection to verify recent influenza infections. It is however unlikely that these vaccinations boosted the CD8^+^ T cell response, as several studies have demonstrated inactivated influenza vaccines are incapable boosting influenza-specific CD8^+^ T cell immunity.^62^

We do recognize that our study had some limitations, the size of our cohort was relatively small and ideally the findings of our study should be confirmed in a larger cohort with timepoints spanning several decades. However, cohorts of longitudinal HLA-typed samples are relatively scarce. Ideally a larger number TCR sequences per donor and visit would have increased the robustness of the TCR repertoire analysis, as it could have allowed us to identify lower frequency TCR clonotypes that are shared between timepoints. The relatively small number of paired TCRαβ sequences in some of our donors/timepoints impeded down sampling across donors. Unfortunately, small blood samples from older individuals and relatively low frequencies of A2/M1_58_-specific CD8^+^ T cells, limited the number of cells that could be index-sorted and sequenced. By depositing our data in publicly available databases, we anticipate that it can be integrated with future dataset for larger longitudinal cohort studies that allow more robust analysis. However, our study confirmed findings from larger cohort studies focussing on age-related changes in the A2/M1_58_-specific CD8^+^ T cell population, namely changes in the paired TCRαβ repertoire of individual donors across age groups of unrelated donors,^28^ or longitudinal studies focussing on TCRβ sequences rather than clonotypes within the same donor over time.^25^ The current study focuses on circulating CD8^+^ T cells and may therefore not reflect changes in the TCR repertoire of other tissue compartments. However, our previous study demonstrated a high level of overlap between the A2/M1_58_-specific TCRαβ repertoire of peripheral blood and tissue compartments.^63^ Ideally, our findings should be confirmed for influenza-specific CD8^+^ T cells with different epitope specificities. Unfortunately, none of the other HLAs was shared among all the donors in our small cohort.

The observations that public A2/M1_58_-specific TCR clonotypes can be maintained for a prolonged period demonstrate a window of opportunity between 30 and 40 years of age, when optimal public TCR and/or public-associated clonotypes potentially could be boosted through vaccinations, so they can be maintained to older age. Recent studies using SARS-CoV-2 vaccine platforms demonstrated that combining different platforms can boost both humoral and cellular immune responses in adults and older individuals.^56^^,^^64^, ^65^, ^66^ If similar vaccine platforms are to be developed for influenza, it would be key to investigate whether these regimens could be combined to boost optimal T cell immunity and preserve public and/or public-associated TCR repertoires for the lifetime.

## Contributors

KK and CES conceptualised the study. KK and CES led the study. CES, MA, THON, SAV, EJG and SS designed and performed the experiments. CES, MA and HAM analysed data. JR and SG provided crucial reagents. CES, THON, SAV, EJG and SS recruited donor cohorts. CES, HAM and KK provided intellectual input into the study design and data interpretation. CES, HAM and KK have accessed and verified the underlying data. CES and KK provided funding. CES and KK wrote the manuscript. All authors reviewed and approved the final version of the manuscript.

## Data sharing statement

TCR sequence data from Supplementary Table S2 has been deposited in Mendeley [https://doi.org/10.17632/Xg79z47zky.1] and VDJdb [https://vdjdb.cdr3.net] and will be available following publication on PubMed. Any additional information required to reanalyse the data in this manuscript is available from the corresponding author upon reasonable request.

## Declaration of interests

HAM consults for Ena Respiratory. The other authors declare no competing interests.

## Appendix ASupplementary data

Supplementary Figs. S1–S8

Van de Sandt_STROBE_checklist
