# Supplementary material for: Gradual changes within long-lived influenza virus-specific CD8+ T cells are associated with the loss of public TCR clonotypes in older adults
Source: eBioMedicine. 2025 Apr 17;115:105697. doi: 10.1016/j.ebiom.2025.105697 (PMC12036069; doi:10.1016/j.ebiom.2025.105697)
Supplement: Supplementary Table S1 [file mmc1.docx]

| **Supplemental Table 1 Longitudinal Cohort Demographics** | | | | | | | | | | | | | | | | | |
| --- | --- | --- | --- | --- | --- | --- | --- | --- | --- | --- | --- | --- | --- | --- | --- | --- | --- |
| **Donor ID** | **Age group** | **Sex** | **Year of Sampling** | **Month sampling** | **Age (years)** | **Timespan (years)** | **Reported Influenza vaccination*** | **HLA-A** | | **HLA-B** | | **HLA-C** | | **Frequency** | **Phenotype** | **TCR** | **Previously published** |
| A2 | Adult | F | 0 | January | 25 | 12 | Annually 2009-2022 | 02:01 | 11:01 | 35:01 | 39:01 | Unk |  | Yes |  | Yes | Valkenburg 2016 PNAS |
|  |  |  | 8 | April | 33 |  |  |  |  |  |  |  |  | Yes | Yes | Yes | van de Sandt 2023 Nature Immunol |
|  |  |  | 12 | April | 37 |  |  |  |  |  |  |  |  | Yes | Yes | Yes |  |
| A11 | Adult | M | 0 | February | 27 | 9 | Unk | 02:01 | 11:01 | 35:01 | 39:01 | Unk |  | Yes |  | Yes | Valkenburg 2016 PNAS |
|  |  |  | 9 | October | 36 |  |  |  |  |  |  |  |  | Yes | Yes | Yes | van de Sandt 2023 Nature Immunol |
| A18 | Adult | F | 0 | May | 41 | 10 | 2009, 2016, 2018* | 02:01 | 03:02 | 18:01 | 35:08 | Unk |  |  |  | Yes | Grant 2016 J Biol Chem |
|  |  |  | 6 | October | 48 |  |  |  |  |  |  |  |  | Yes | Yes | Yes | van de Sandt 2023 Nature Immunol |
|  |  |  | 10 | November | 52 |  |  |  |  |  |  |  |  | Yes | Yes | Yes |  |
| OA3 | Older Adult | F | 0 | December | 73 | 10 | Annually 2009-2023 | 02:01 |  | 07:02 | 44:02 | 05:01 | 07:02 | Yes | Yes | Yes |  |
|  |  |  | 6 | January | 79 |  |  |  |  |  |  |  |  | Yes | Yes | Yes | van de Sandt 2023 Nature Immunol |
|  |  |  | 10 | January | 83 |  |  |  |  |  |  |  |  | Yes | Yes | Yes |  |
| OA18 | Older Adult | F | 0 | October | 66 | 9 | Annually 2009-2023 | 02:01 |  | 15:01 | 44:02 | Unk |  | Yes |  | Yes | Nguyen 2017 JLB |
|  |  |  | 5 | May | 71 |  |  |  |  |  |  |  |  | Yes | Yes | Yes | van de Sandt 2023 Nature Immunol |
|  |  |  | 9 | November | 75 |  |  |  |  |  |  |  |  | Yes | Yes | Yes |  |
| OA19 | Older Adult | F | 0 | November | 83 | 9 | Annually 2009-2013 | 01:01 | 02:01 | 08:01 | 13:02 | Unk |  | Yes | Yes | Yes |  |
|  |  |  | 5 | July | 88 |  |  |  |  |  |  |  |  | Yes | Yes | Yes | van de Sandt 2023 Nature Immunol |
|  |  |  | 9 | January | 92 |  |  |  |  |  |  |  |  | Yes | Yes | Yes |  |
| OA31 | Older Adult | M | 0 | July | 61 | 7 | Annually 2015-2022 | 02:01 | 03:01 | 07:02 | 50:01 | 06:02 | 07:02 | Yes |  | Yes | Nguyen 2017 JLB |
|  |  |  | 3 | January | 64 |  |  |  |  |  |  |  |  | Yes | Yes | Yes | van de Sandt 2023 Nature Immunol |
|  |  |  | 7 | November | 68 |  |  |  |  |  |  |  |  | Yes | Yes | Yes |  |
| Unk=Unknown *These were years reported to us, we cannot exclude that donors were vaccinated in other years | | | | | | | | | | | | | | | | | |
