# Supplementary material for: Gradual changes within long-lived influenza virus-specific CD8+ T cells are associated with the loss of public TCR clonotypes in older adults
Source: eBioMedicine. 2025 Apr 17;115:105697. doi: 10.1016/j.ebiom.2025.105697 (PMC12036069; doi:10.1016/j.ebiom.2025.105697)
Supplement: Supplementary Figs. S1–S8 [file mmc3.pdf]

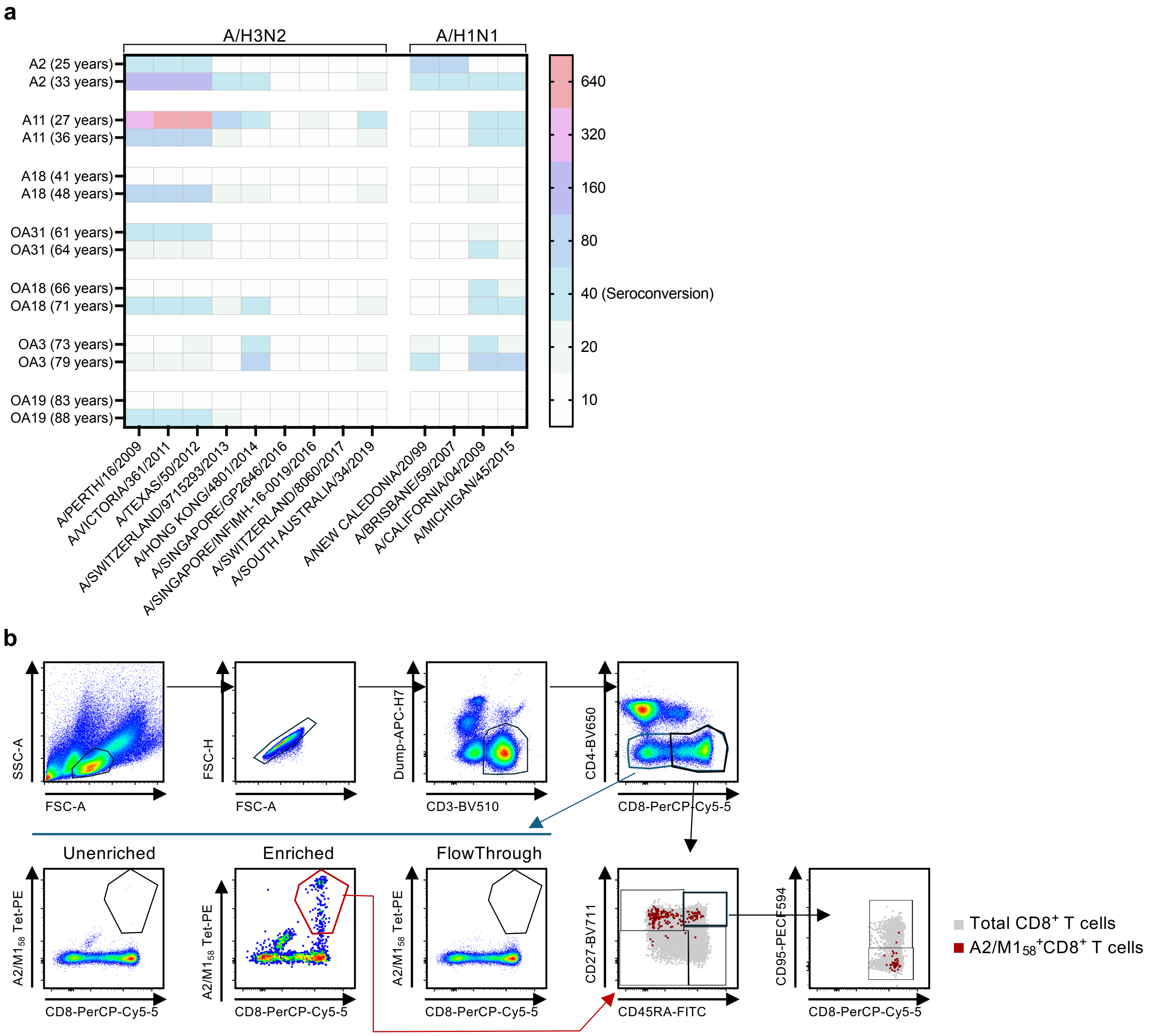

Supplementary Fig. 1 Infection history and gating strategy A2/M1$_{58\text{-}66}$$^+$CD8$^+$ T cells.
a) Influenza HI antibody titres against historical viruses in the HLA-A*02:01 participants. **b)** Representative FACS panels for the gating strategy used to characterize the total CD8$^+$ T cell and the A2/M1$_{58}$$^+$CD8$^+$ T cell populations, including the unenriched, enriched and flowthrough fractions of the TAME assay. The unenriched fraction was used to define the frequency and phenotype of the total CD8$^+$ T cell population (grey cell populations), whereas the A2/M1$_{58}$ tetramer-positive CD8$^+$ T cells of the enriched fraction were used to define the frequency and phenotype of the A2/M1$_{58}$$^+$CD8$^+$ T cell population (red gate and cell populations). Naïve and memory T cell subsets were identified as T_{cm} (CD27$^+$CD45RA$^-$) cells, T_{em} (CD27$^-$CD45RA$^-$), T_{emra} (CD27$^-$CD45RA$^+$), T_{scm} (CD27$^+$CD45RA$^+$CD95$^+$) and $T_{naïve}$ (CD27$^+$CD45RA$^+$CD95$^-$) or as $T_{naïve\text{-}like}$ (CD27$^+$CD45RA$^+$) cells.

a

Donor	Total number of paired TCR$\alpha\beta$ V1	V2	V3
All adults	76	99	43
All older adults	95	112	77
A2	26	46	16
A11	24	26	
A18	26	27	27
OA31	38	19	21
OA18	20	52	24
OA3	14	14	23
OA19	23	27	9

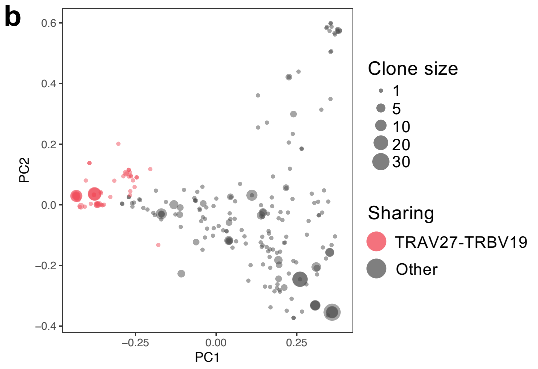

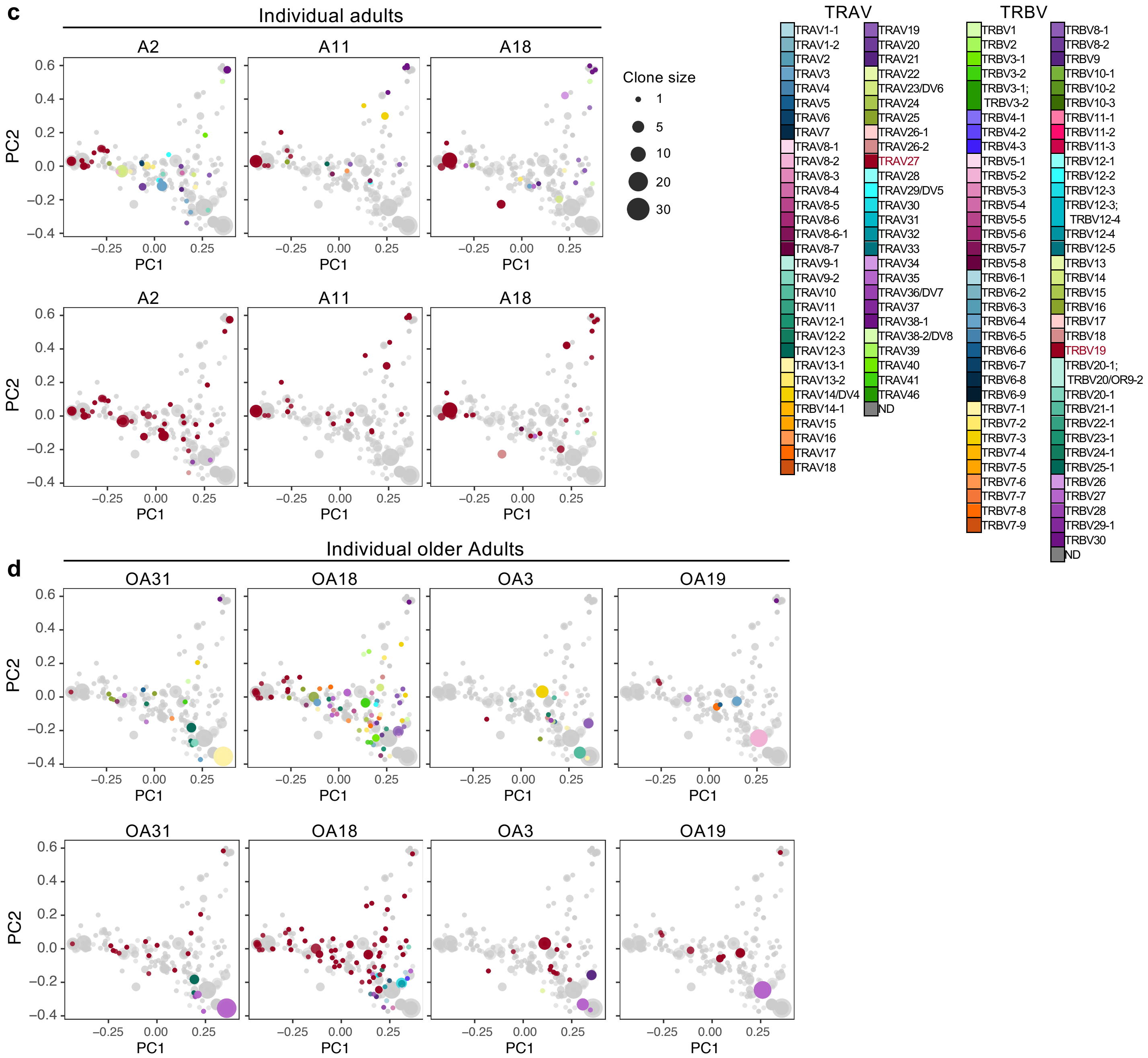

Supplementary Fig. 2 TRAV and TRBV gene usage in the A2/M1$_{58\text{-}66}$$^{+}$CD8^{+} TCR$\alpha\beta$ repertoire of individual donors.

TCR$\alpha\beta$ analysis of single-cell sorted enriched A2/M1$_{58}$$^{+}$CD8^{+} T cells. **a)** Total number of paired$\alpha\beta$ clonotypes per donor per timepoint. TCRdist generated 2D kernel principal components analysis (PCA) projection of the A2/M1$_{58}$$^{+}$CD8^{+} TCR landscape **b)** for all donors combined (n=7), TRAV27-TRBV19 paired TCR clonotypes coloured in red or **c-d)** within each donor colored by Vα (top), and Vβ (bottom) gene usage, the full repertoire of all donors and time points combined is indicated in grey at the background. Clone size indicated by symbol size.

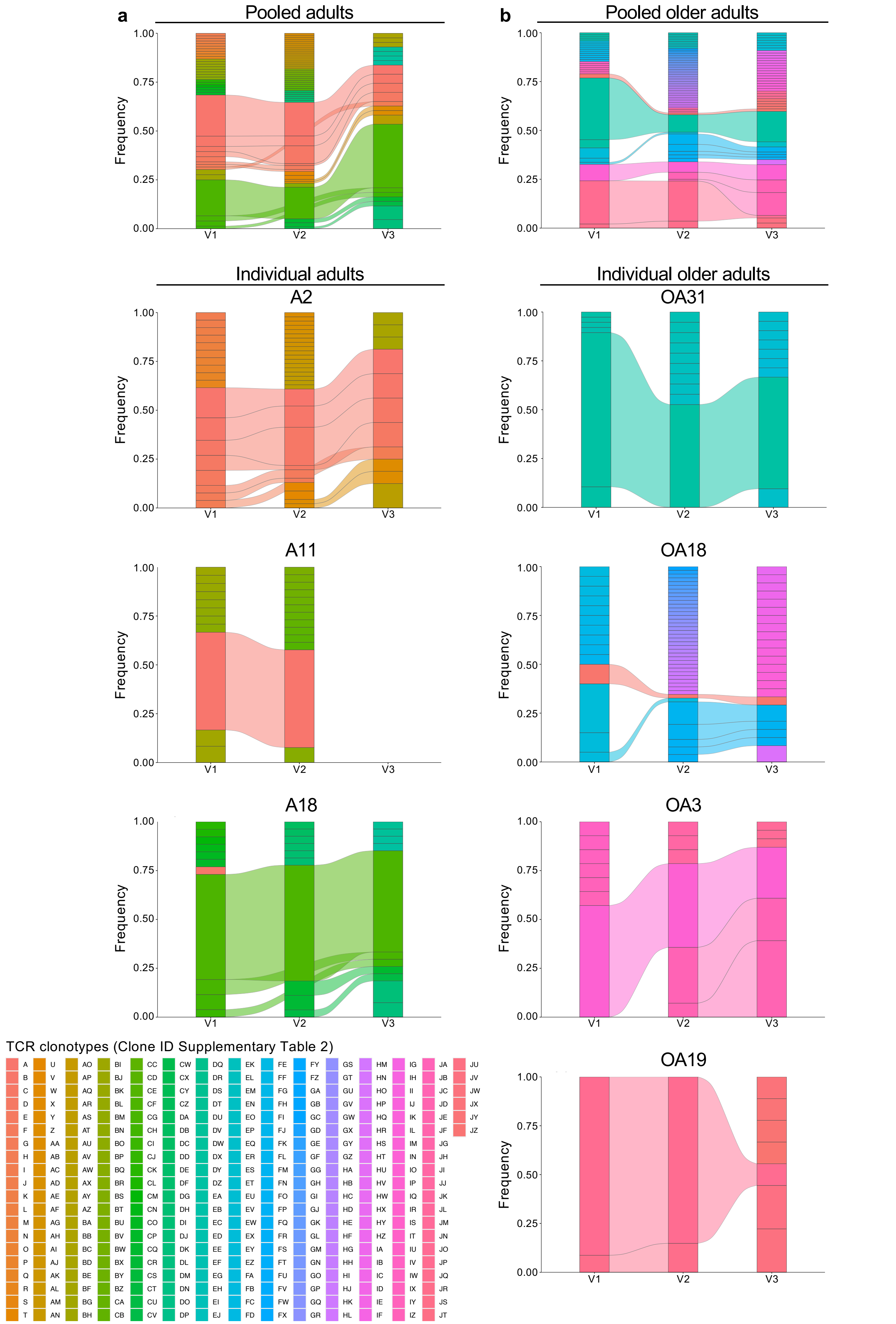

Supplemental Figure 3

van de Sandt *et al*

Supplementary Fig. 3. Longitudinal persistence of paired A2/M1$_{58}$$^{+}$CD8^{+} TCRαβ clonotypes.

Frequency and persistence of paired A2/M1$_{58}$$^{+}$CD8^{+} TCRαβ clonotypes across the three timepoints (V1, V2, V3) for **a)** pooled (n=3) and individual adult donors and **b)** pooled (n=4) and individual older adult donors. Shared clonotypes are connected by coloured lines. TCR clonotypes are labelled according to the clone ID in Supplementary Table 2.

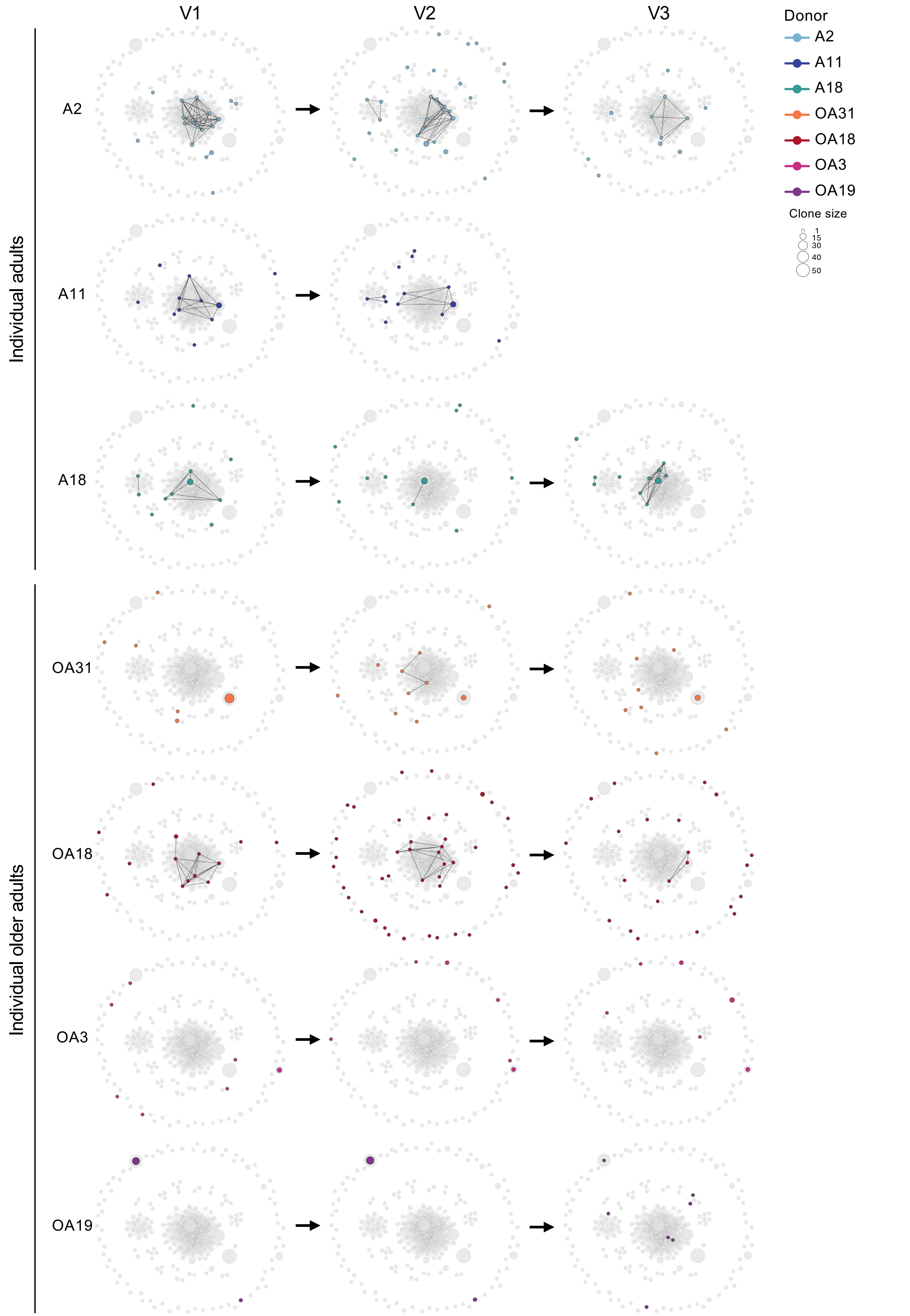

Supplemental Figure 4

van de Sandt *et al*

Supplementary Fig. 4. Shift from high similarity among A2/M1$_{58}$$^{+}$CD8^{+} TCRαβ clonotypes in adults to low similarity in older adults.

Similarity network analysis of A2/M1$_{58}$$^{+}$CD8^{+} TCRαβ clonotypes of individual donors across time. Clonotypes of individual donors shown in respective donor colour, larger network (Fig. 4a) depicted in light grey. Each node of the similarity network is a uniquely paired TCRαβ sequence, edges connect TCRαβ with TCRdist less than or equal to 120. Coloured by TCRα-motifs (left) and TCRβ-motifs (right). Single clonotypes without connections are depicted in the outer circle.

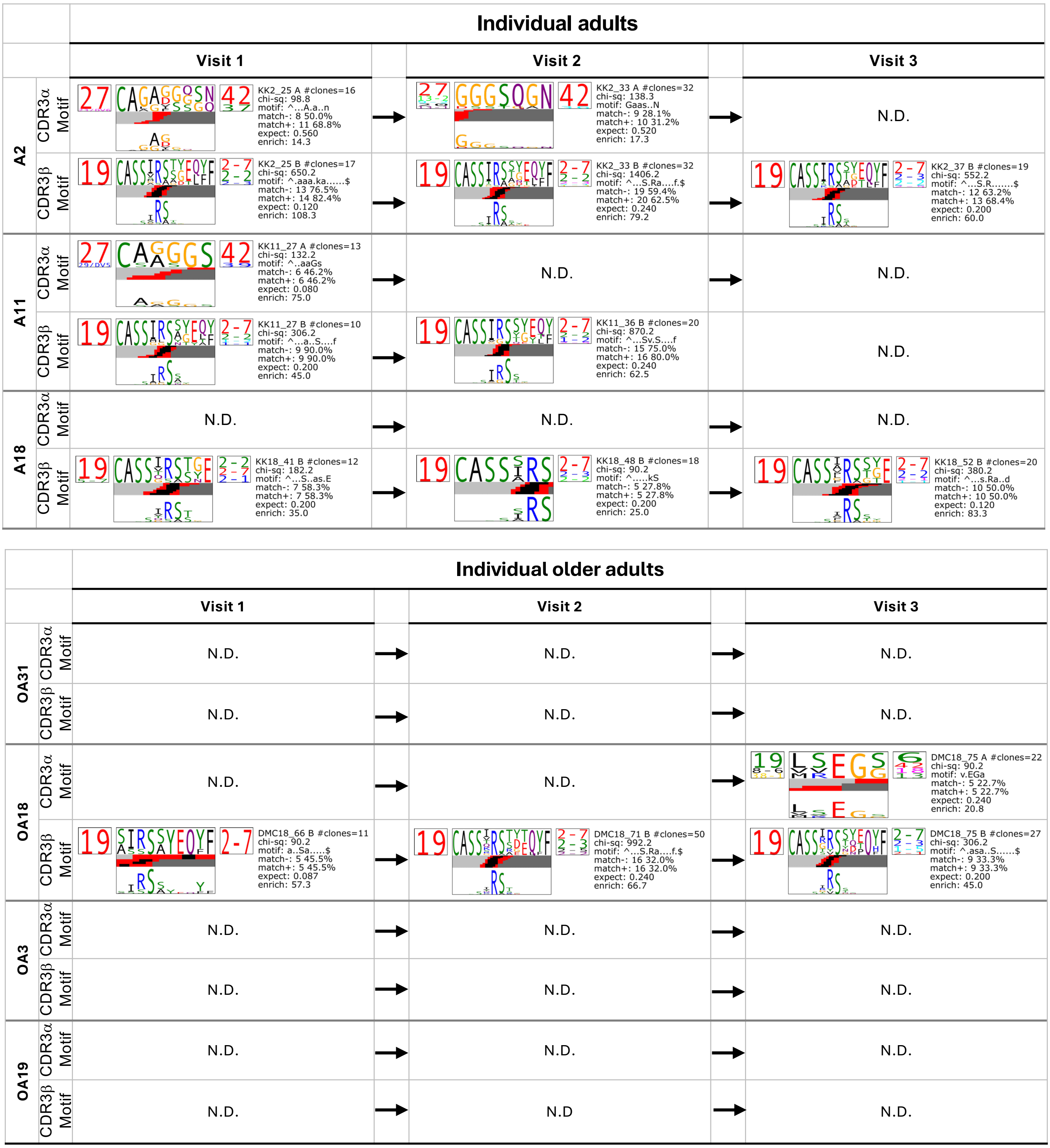

Supplementary Fig. 5. Public A2/M1$_{58}$$^{+}$CD8^{+} CDR3β motifs stably expressed among adult donors.

a) Top-scoring A2/M1$_{58}$$^{+}$CD8^{+} CDR3α and CDR3β logo sequence motifs for each donor across timepoints (visits). Each logo depicts the V- (left side) and J- (right side) gene frequencies with the CDR3 amino acid sequence in the middle with the full height (top) and scaled (bottom) by per-residue reparative entropy to background frequencies derived from TCRs with matching gene-segment composition to highlight motif positions under selection. The middle section indicates the inferred rearrangement structure by source region (light grey for V-region, dark grey for J, black for D and red for N-insertions) of the grouped receptors.

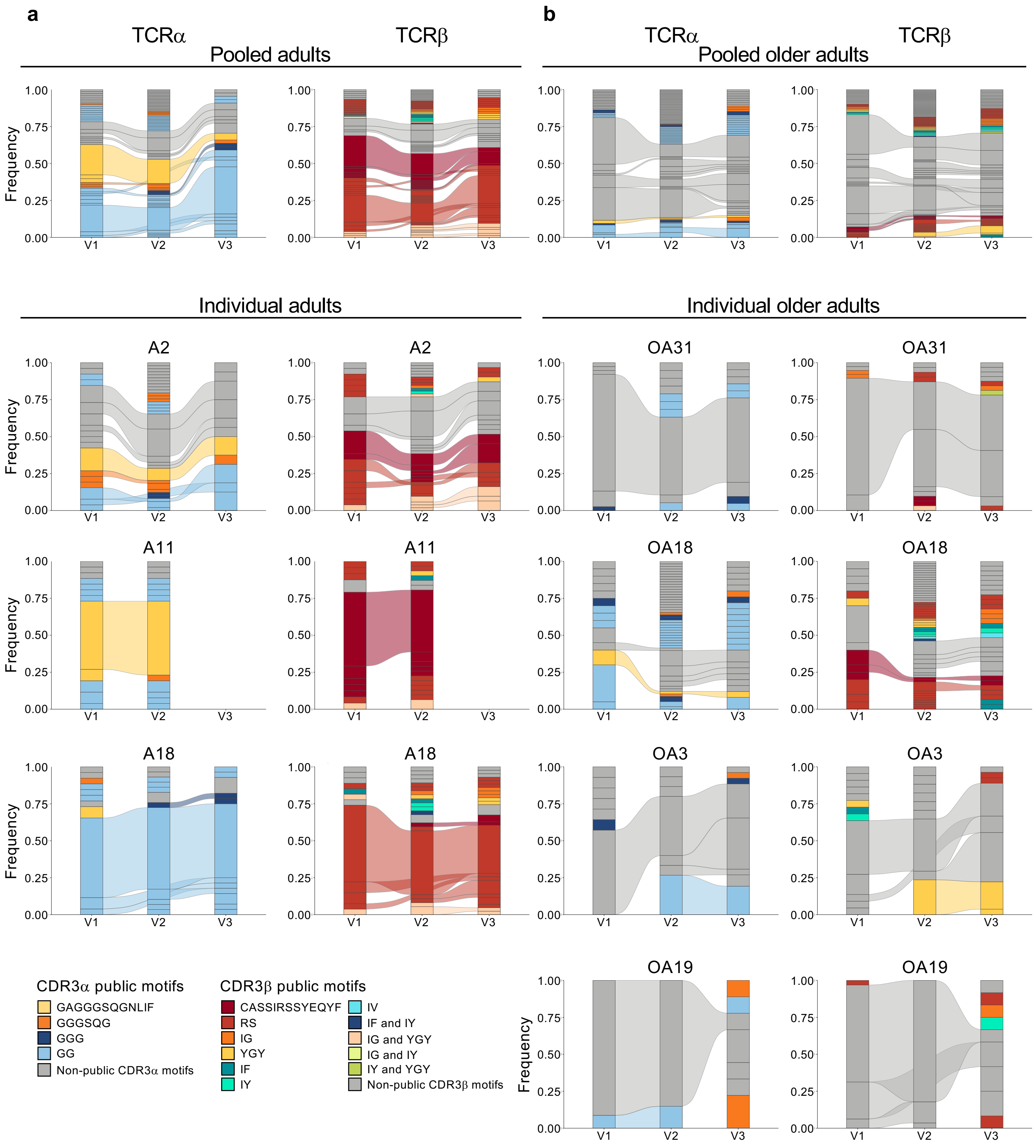

Supplementary Fig. 6. Longitudinal changes A2/M1$_{58}$$^{+}$CD8^{+} CDR3 motifs.
Frequency and persistence of A2/M1$_{58}$$^{+}$CD8^{+} CDR3α (left) and CDR3β (right) motifs across the three timepoints (V1, V2, V3) for **a)** pooled (n=3) and individual adult donors and **b)** pooled (n=4) and individual older adult donors. Shared clonotypes are connected by coloured lines.

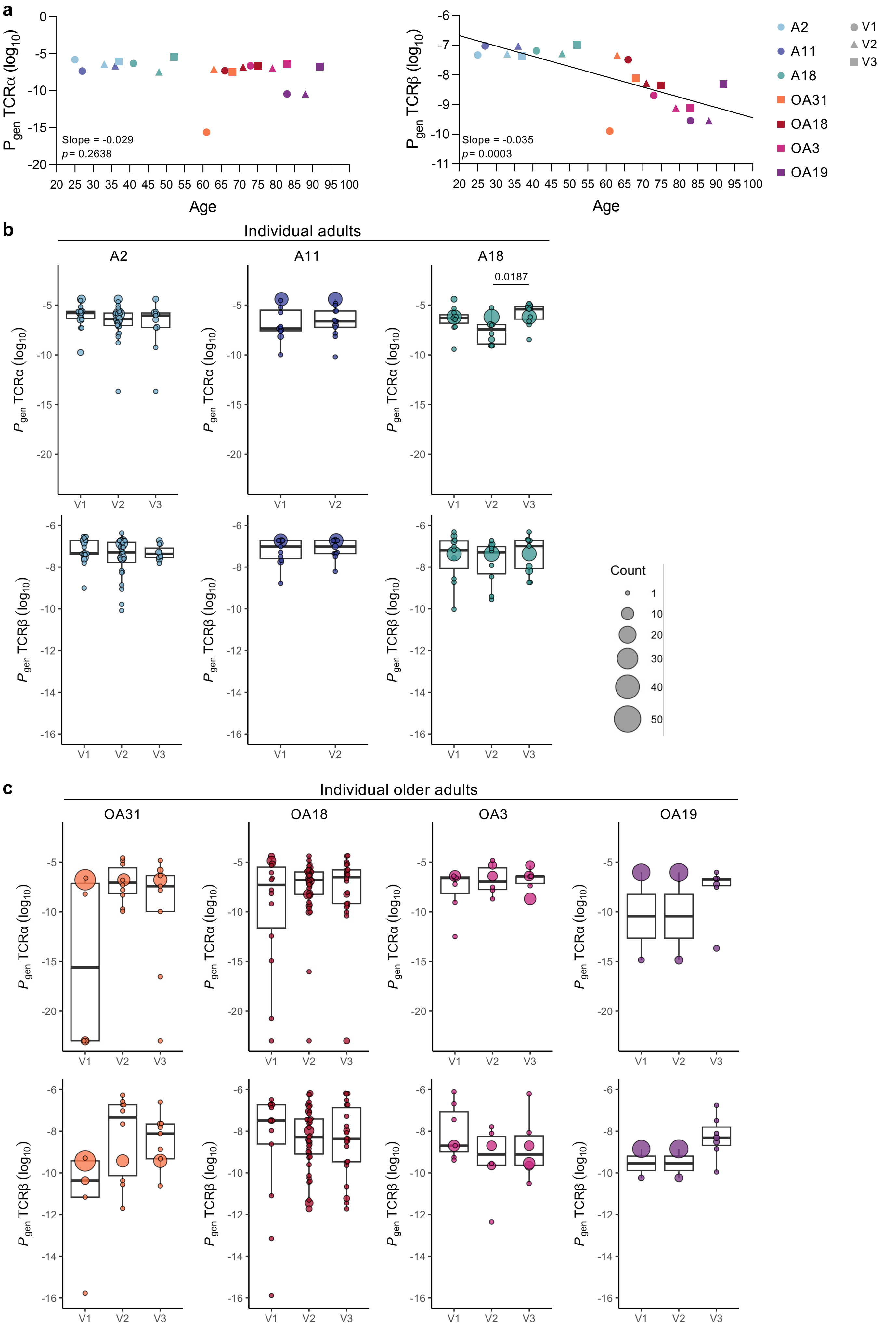

Supplemental Figure 7

van de Sandt *et al*

Supplementary Fig. 7. Longitudinal changes in probability of generation within the A2/M1$_{58}$$^{+}$CD8^{+} TCRs

Correlation of age with probabilities of generation (P_{gen}; log10 transformed) for all single **a)** TCRα and **b)** TCRβ chains of each donor and timepoint estimated with TCRdist. Correlation was established by using the Spearman's rank correlation (r_s), *p*-values are indicated in the left bottom corner of the graphs. Probabilities of generation (P_{gen}; log10 transformed) in individual **b)** adults and **c)** older adults for all single TCRα (top) and TCRβ (bottom) chains proteins across all three timepoints estimated with TCRdist. Box plots represent the median (middle bar), 75% quartile (upper hinge) and 25% quartile (lower hinge) with whiskers extending 1.5 times the inter-quartile range, dots represent individual clonotypes present at each timepoint. Clone size indicated by symbol size. Statistical analysis of P_{gen} utilized a two-sided Kruskal-Wallis with Dunn's correction for multiple tests between timepoints. *p*-values are indicated above the graphs.

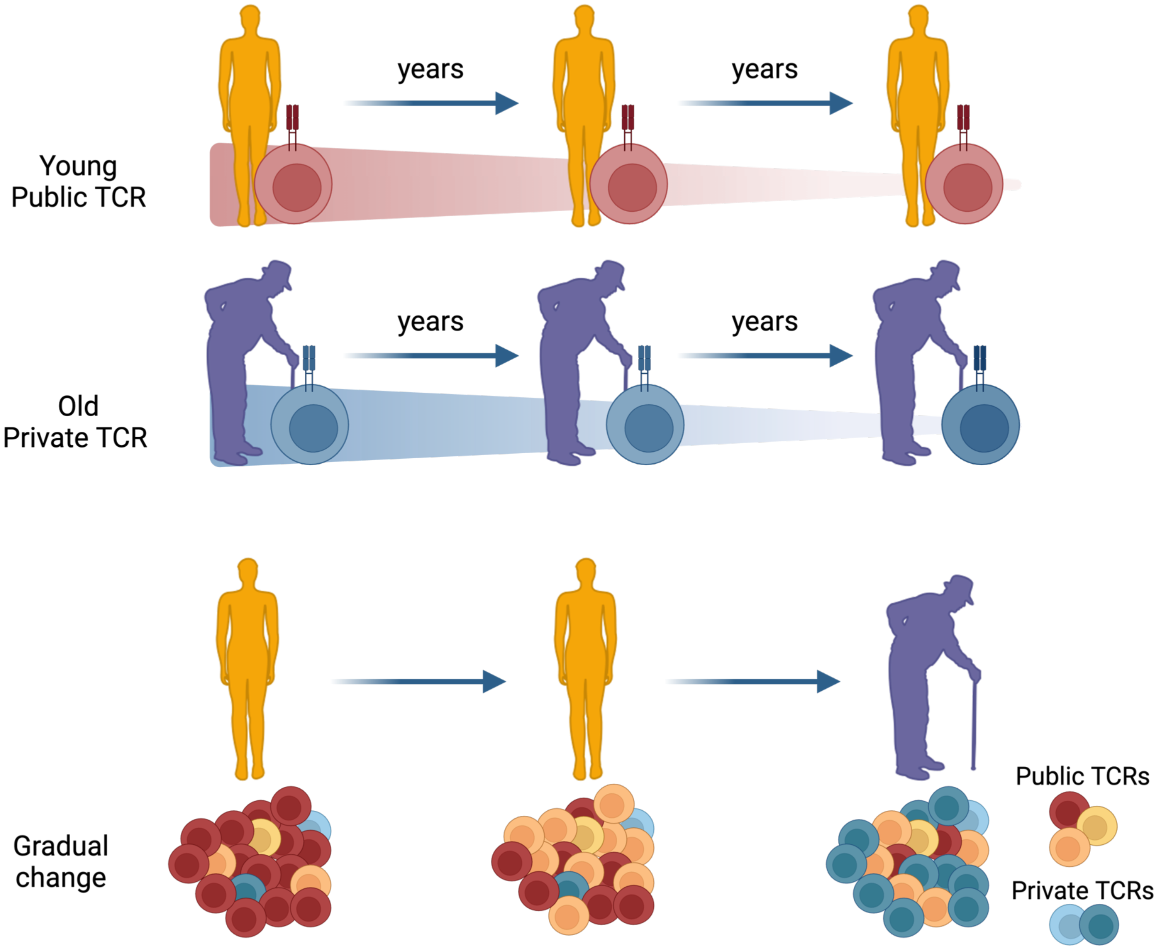

Supplementary Fig. 8. Graphical summary
The clonotype switch observed from adults to older adults, resulted from a gradual decline of public clonotypes in adults, which was initially compensated by expansion of closely related clonotypes expressing public-associated features. Once these public-associated TCR clonotypes were abated in older adults, the void was filled by expansions of private TCR clonotypes, which shared less similarity with young clonotypes. Expanded private clonotypes in older adults also declined over time and were gradually replaced by other private clonotypes with low similarity to the public clonotypes observed in adults
